# Positional relationships between a tracheal diverticulum and the tracheal tube under general anesthesia: a single-center observational and simulation study

**DOI:** 10.1186/s12871-023-02347-y

**Published:** 2023-11-25

**Authors:** Kunihiro Mitsuzawa, Tsukasa Kumagai, Haruo Uchida, Toshiyuki Shimizu

**Affiliations:** 1 Department of Anesthesiology and Resuscitology, Shinshu University School of Medicine, 3-1-1, Asahi, Matsumoto City, Nagano, 390-8621 Japan; 2 Department of Anesthesiology, Nagano Prefectural Shinshu Medical Center, 1332, Suzaka City, Nagano, 382-8577 Japan

**Keywords:** Bronchial intubation, Malposition of tracheal tube, Tracheal diverticulum, Tracheal intubation, Tracheal tube

## Abstract

**Background:**

Incomplete sealing of tracheal diverticula by a tracheal tube cuff during positive-pressure ventilation causes barotrauma but the concrete possibility of incomplete sealing has not been indicated.

We aimed to assess the possibility of incomplete sealing in a simulated situation of tracheal intubation for patients with tracheal diverticula with tube fixation where the tracheal tube’s vocal cord guide overlaps with the patient’s vocal cord.

**Methods:**

We retrospectively assessed the characteristics of tracheal diverticula based on thoracic computed tomography data in our institution from January 2018 to July 2020. Then, we assessed the structural parameters of three single-lumen tracheal tubes (Parker Flex-Tip [Parker Medical, Bridgewater, CT, USA], Portex Soft Seal [ICU Medical, San Clemente, CA, USA], and Shiley TaperGuard [Medtronic, Dublin, Ireland]; 6.0–8.0 mm inner diameter size) and simulated the positional relationships between tracheal diverticula and the tracheal tube during tracheal intubation where the vocal cord guide overlaps with the patient’s vocal cord. We assessed each tube product’s possibility of incompletely sealing tracheal diverticula and the possibility of unintended bronchial intubation.

**Results:**

In 5,854 patients, the prevalence of tracheal diverticula was 5.7%. The mean (SD) length from the vocal cord to the distal end of the tracheal diverticula was 52.2 (12.8) mm. Tracheal tubes with length from the distal end of the tracheal cuff to the vocal cord guide of ≥ 70 mm had a low risk of incompletely sealing tracheal diverticula (< 5%) and length from the distal end of the tube to the vocal cord guide of ≤ 95 mm had a low risk of unintended bronchial intubation (< 5%). No products in this study satisfied both outcomes.

**Conclusions:**

Tube fixation, where the vocal cord guide overlaps with the patient’s vocal cord, is associated with risk of incompletely sealing of tracheal diverticula depending on the tube’s manufacturer and tube’s inner diameter size, although it was not a high risk. The use of small inner diameter sized tube relative to patient’s body size is high risk of incomplete sealing of tracheal diverticula.

**Trial registration:**

This trial was prospectively registered at University Hospital Medical Information Network (UMIN). Clinical trial number and registry URL: UMIN000043317 (URL: https://center6.umin.ac.jp/cgi-open-bin/ctr_e/ctr_view.cgi?recptno=R000048055).

**Supplementary Information:**

The online version contains supplementary material available at 10.1186/s12871-023-02347-y.

## Introduction

**Fig. 1:**
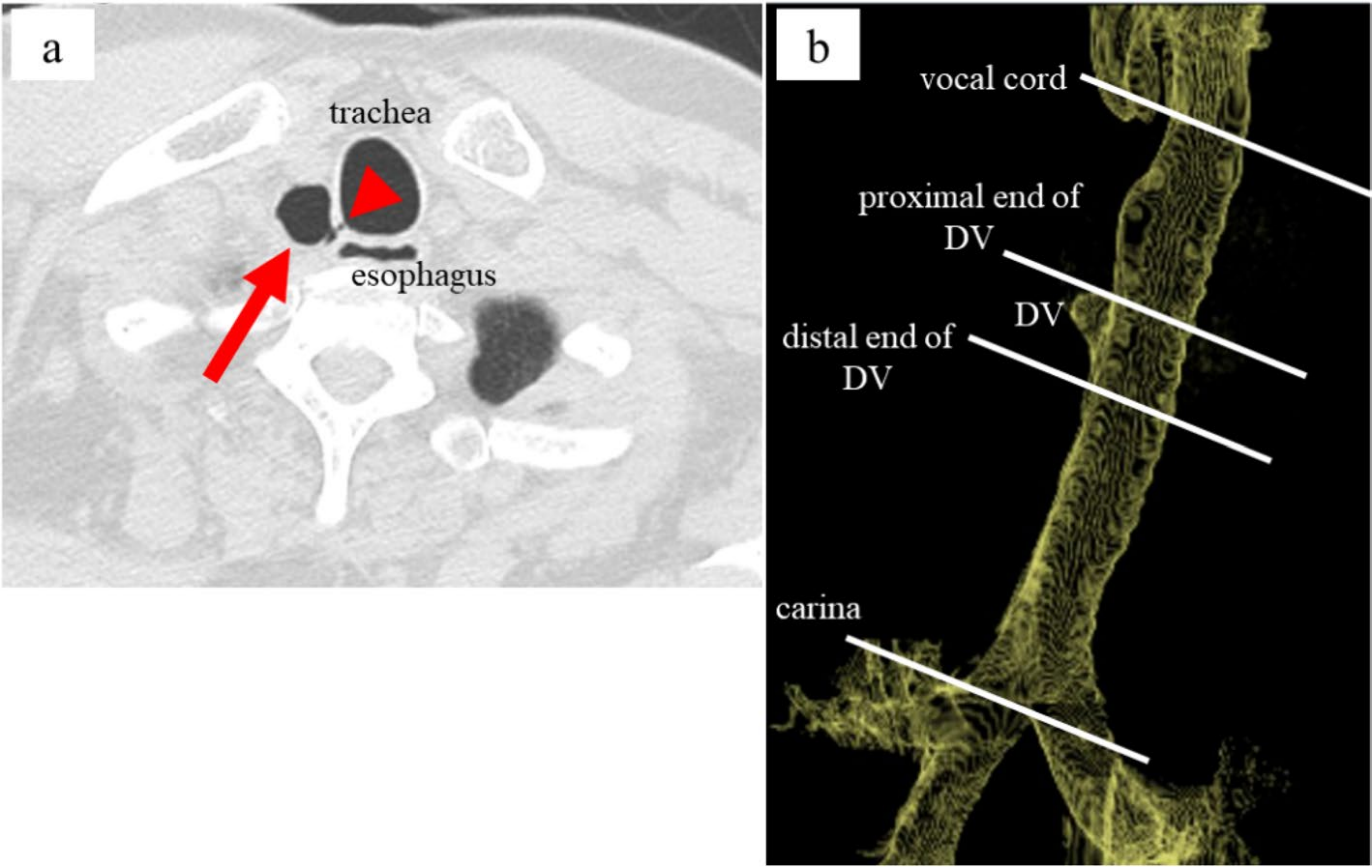
An example of CT image of the tracheal diverticulum and trachea in this study. **a** Axial view of chest CT image of the tracheal diverticulum (arrow) with an evident orifice (arrowhead). **b** 3D-CT image of tracheal diverticulum, trachea and anatomical sites relevant to this study. DV: tracheal diverticulum

Tracheal diverticulum is a form of paratracheal air cyst. A direct connection with the trachea is visible on CT in 8–35% of cases. This connection makes a paratracheal cyst a diverticulum (Fig. 1) [1–4]. However, a dynamic CT report has shown that even in paratracheal air cysts without an evident orifice, the cyst contracts during inspiration and expands during expiration, indicating that the many paratracheal air cysts without an evident orifice might be tracheal diverticula and are actually in communication with the trachea through hidden orifices [2]. In general anesthesia, tracheal diverticula could cause failures in tracheal intubation, positive-pressure ventilation, and lung isolation [5–7]. Additionally, positive-pressure ventilation for tracheal diverticula could causes tracheal diverticula rupture, subcutaneous emphysema, and pneumomediastinum [7–14]. Thus, preventing positive-pressure ventilation to tracheal diverticula is important to avoid these adverse events.

The method to prevent these events involves the complete sealing of tracheal diverticula by the tracheal cuff, which is located distal to tracheal diverticula, such as deeply intubated single-lumen tracheal tube or double-lumen tracheal tube [15]. Single-lumen tracheal tubes with tube fixation where the vocal cord guide of the tracheal tube overlaps with the patient’s vocal cord are commonly used. Moreover, the structural design and inner diameter size of tracheal tubes differ across various manufacturers [16]. Thus, the positional relationships between tracheal diverticula and the tracheal tube and the possibility of the incomplete sealing of tracheal diverticula are not well known.

This study aimed to assess the possibility of incomplete sealing of a tracheal diverticulum by the tracheal cuff of a single-lumen tracheal tube during tracheal intubation for patients with tracheal diverticula. We hypothesized that the fixation of the tracheal tube such that the vocal cord guide of the tracheal tube overlaps with the patient’s vocal cord for these patients may cause incomplete sealing of tracheal diverticula. Accordingly, we investigated the characteristics of tracheal diverticula and assessed the positional relationships between tracheal diverticula and the tracheal tube during tracheal intubation.

## Methods

This study comprises two substudies: (1) investigation of the characteristics of tracheal diverticula and (2) investigation of the structural parameters of the tracheal tube and positional relationships between tracheal diverticula and the tracheal tube. Substudy 1 (a retrospective observational study) was approved by Shinshu Medical Center Institutional Review Board (IRB #2–23) on 05/10/2020, and registered at the University hospital Medical Information Network (UMIN) Clinical Trials (UMIN000043317) on 15/02/2021 (registry URL: https://center6.umin.ac.jp/cgi-open-bin/ctr_e/ctr_view.cgi?recptno=R000048055). Kunihiro Mitsuzawa, the first author of this paper, was the principal investigator. The study was conducted at the Shinshu Medical Center, Suzaka, Japan. The participant recruitment began on 15/02/2021 and the study was completed on 20/03/2021. Because this was a retrospective study and it was not able to provide informed consent to all participants in this study, Shinshu Medical Center Institutional Review Board waived the requirement for individual written informed consent. Study information was available on the hospital website and hospital bulletin board allowing participants the opportunity to opt‐out. All the procedures were followed in accordance with Declaration of Helsinki and recommended Strengthening the Reporting of Observational Studies in Epidemiology (STROBE) guidelines.

### Substudy 1: characteristics of tracheal diverticula

All patients who underwent thoracic CT in our institution between January 2018 and July 2020 were included in this study. The indication of thoracic CT was not confined. For patients who underwent thoracic CT two or more times (duplication of patients), the most recent CT data were used. The following patients were excluded: (1) patients whose CT data did not include whole lengths of the trachea and (2) patients whose CT slice thickness was ≥ 3 mm to avoid overlooking tracheal diverticula.

**Fig. 2:**
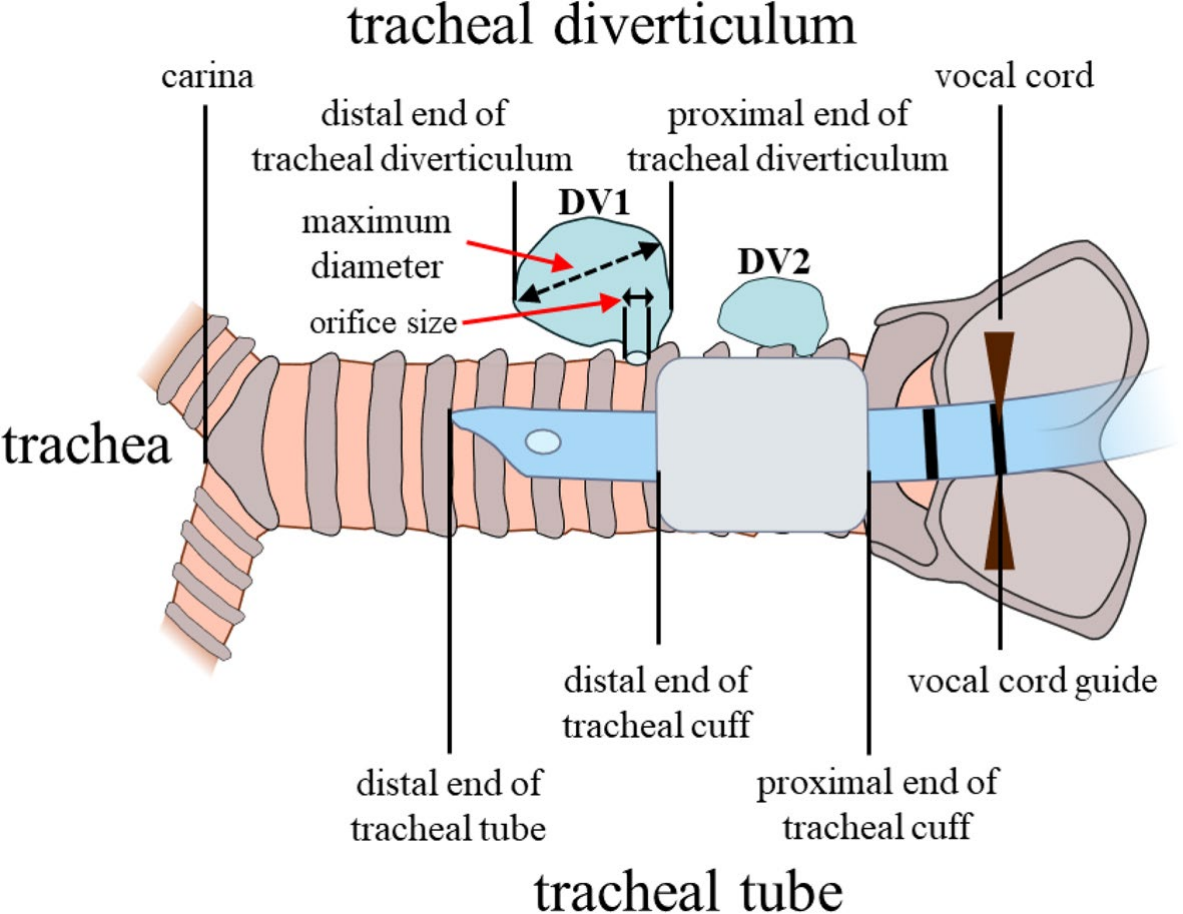
The scheme of simulated positional relationships between tracheal diverticula and the tracheal tube during tracheal intubation where the vocal cord guide overlaps with the patient’s vocal cord and characteristics of the trachea, tracheal diverticula and tracheal tubes assessed in this study. DV1: tracheal diverticulum which is located distal to the distal end of tracheal cuff and at risk of receiving positive-pressure ventilation. DV2: tracheal diverticulum which is located proximal to the distal end of tracheal cuff and prevented from receiving positive-pressure ventilation

The primary outcome was the prevalence of tracheal diverticula. The secondary outcomes were the characteristics of tracheal diverticula, including the number of tracheal diverticula; maximum size of tracheal diverticula; the prevalence of an evident orifice of the trachea; size of the orifice; lengths from the vocal cord to the proximal end of tracheal diverticula, from the vocal cord to the distal end of tracheal diverticula, and from the distal end of tracheal diverticula to the carina; and tracheal length (length from the vocal cord to the carina) (Fig. 2). Radiologic interpretations were performed by two anesthesiologists trained by a thoracic surgeon who was familiar with tracheal diverticula. In the case of disagreements in interpretations between these anesthesiologists, the thoracic surgeon performed interpretation. All of the chest CTs in this study were performed while maintaining deep inspiration, and the presence of respiratory variability in the paratracheal air cyst which suggests the communication with trachea could not be confirmed. Therefore, we considered that all of the paratracheal air cysts in this study were tracheal diverticula in communication with the trachea and all of the paratracheal air cysts in this study were included in the analysis. Furthermore, even in tracheal diverticulum with an evident orifice, the possibility of having other hidden orifices cannot be ruled out. To prevent barotrauma to the tracheal diverticulum during positive pressure ventilation in a clinical setting, it is important to seal and bypass not only the obvious orifice but also the entire tracheal diverticulum by the tracheal tube because the positional relationships between all of the orifices, including hidden orifices, and the trachea are not known. Hence, we analyzed the positional relationship between the diverticulum and the trachea, which is necessary to seal the entire tracheal diverticulum, and did not analyze the positional relationship between the evident orifice and the trachea.

### Substudy 2: structural parameters of the tracheal tube and positional relationships between tracheal diverticula and the tracheal tube

The structural parameters of three single-lumen tracheal tubes (five different inner diameter sizes: 6.0–8.0 mm) in Japan (Parker Flex-Tip, Parker Medical, Bridgewater, CT, USA; Portex Soft Seal, ICU Medical, San Clemente, CA, USA; Shiley TaperGuard, Medtronic, Dublin, Ireland) were measured (Supplementary Fig. 1): lengths from the distal end of the tracheal tube to the distal end of the tracheal cuff, from the distal end of the tracheal tube to the proximal end of the tracheal cuff, from the distal end of the tracheal cuff to the vocal cord guide, and from the distal end of the tracheal tube to the vocal cord guide (Fig. 2). Then, the positional relationship between tracheal diverticula and the tracheal tube was simulated in the situation of tracheal intubation. We assessed the percentage of tracheal diverticula located distal to the tracheal cuff of the fixed tracheal tube such that the vocal cord guide overlapped with the patient’s vocal cord as a risk of incomplete sealing of tracheal diverticula.

### Statistical analysis

We performed Student's t-test to compare the two groups and Fisher's exact test to analyze 2 × 2 contingency tables. For each test, a two-sided *P*-value of < 0.05 was considered statistically significant. Statistical analyses were performed with R version 3.4.1 (R Foundation for Statistical Computing, Austria). We performed power analysis for Substudy 1. We assumed the prevalence of tracheal diverticula in this study as 3.0% based on previous studies.^14–20^ The width of 95% confidence interval (CI) of prevalence of tracheal diverticulum was assumed as 1.0% and 4,472 CT scans were required for final analysis as the minimum sample size. Thus, we determined the enrolment period of this study according to the number of CT scans needed for final analysis and the annual number of patients who underwent CT in our institution.

## Results

### Substudy 1: characteristics of tracheal diverticula

**Fig. 3:**
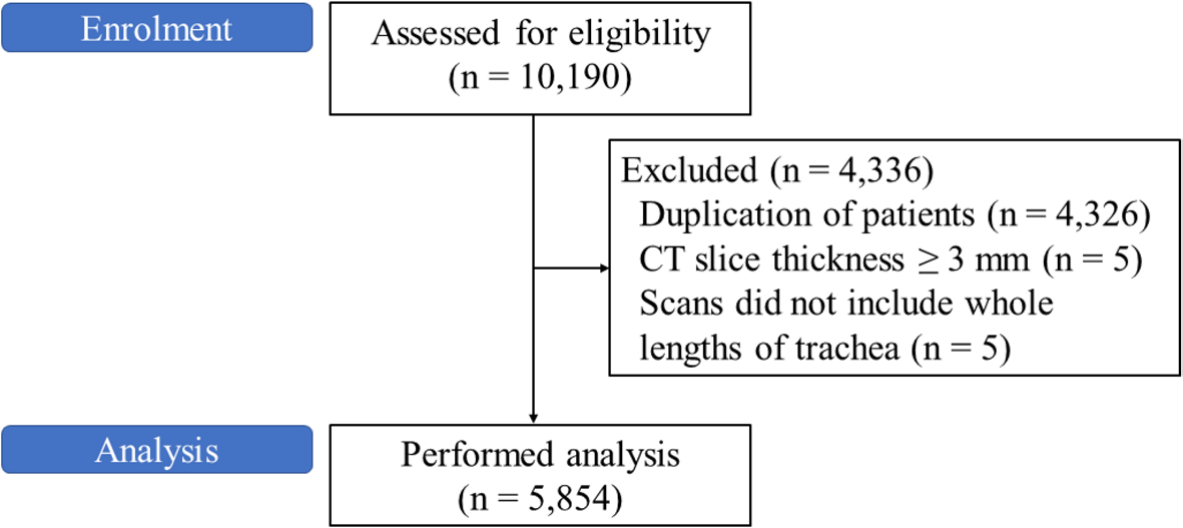
Flow diagram of this study. CT, computed tomography

During the enrolment period, 10,190 CT scans were assessed for eligibility. In total, 4,326 CT scans were excluded because of duplication, 5 because of slice thickness being ≥ 3 mm, and 5 because of the lack of imaging of the whole length of the trachea. Finally, 5,854 CT scans were included in the final analysis (Fig. 3). The mean (standard deviation [SD]) age of the included patients was 71.1 (17.5) years, and 51.9% were males. All patients were Asian.

In total, 361 tracheal diverticula were found in 333 patients. The prevalence of tracheal diverticula was 5.7% (95% CI 5.1%–6.3%). The mean age was not significantly different between patients with tracheal diverticula and those without (mean (SD): 71.2 (16.1) vs 71.1 (17.6), *p* = 0.902). Age-stratified prevalence was similar among the age groups (Supplementary Table 1). The ratio of females was higher in patients with tracheal diverticula than in those without (54.4% vs 47.8%, *p* = 0.0206). The median number of tracheal diverticula was 1 (range: 1–4), and 38.2% of tracheal diverticula had evident orifice.

The characteristics of tracheal diverticula are shown in Table 1. The maximum diameter of tracheal diverticula ranged from 2.0 to 44.0 mm [mean (SD) = 8.3 (6.2) mm]. The mean (SD) lengths from the vocal cord to the proximal end of tracheal diverticula and from the vocal cord to the distal end of tracheal diverticula were 44.5 (14.0) mm and 52.2 (12.8) mm, respectively. The tracheal length ranged from 86 to 162 mm [mean (SD) = 120.5 (12.8) mm]. Tracheal lengths were ≤ 90 mm in two (0.6%) patients. Tracheal diverticula were commonly located on the right dorsal side of the trachea at the sternoclavicular joint level (89.5%), which almost coincided with the level of the second thoracic vertebral body.

**Table 1:** Anatomical properties of tracheal diverticula

Maximum diameter (mm)	8.3 [6.2]
Orifice size (mm)	3.6 [3.0]
Vocal cord-to-proximal end of tracheal diverticulum (mm)	44.5 [14.0]
Vocal cord-to-distal end of tracheal diverticulum (mm)	52.2 [12.8]
Distal end of tracheal diverticulum-to-carina (mm)	68.2 [12.4]
Tracheal length (vocal cord-to-carina) (mm)	120.5 [12.8]
Values are presented as mean [SD]	

### Substudy 2: structural parameters of the tracheal tube and positional relationships between tracheal diverticula and the tracheal tube

**Fig. 4:**
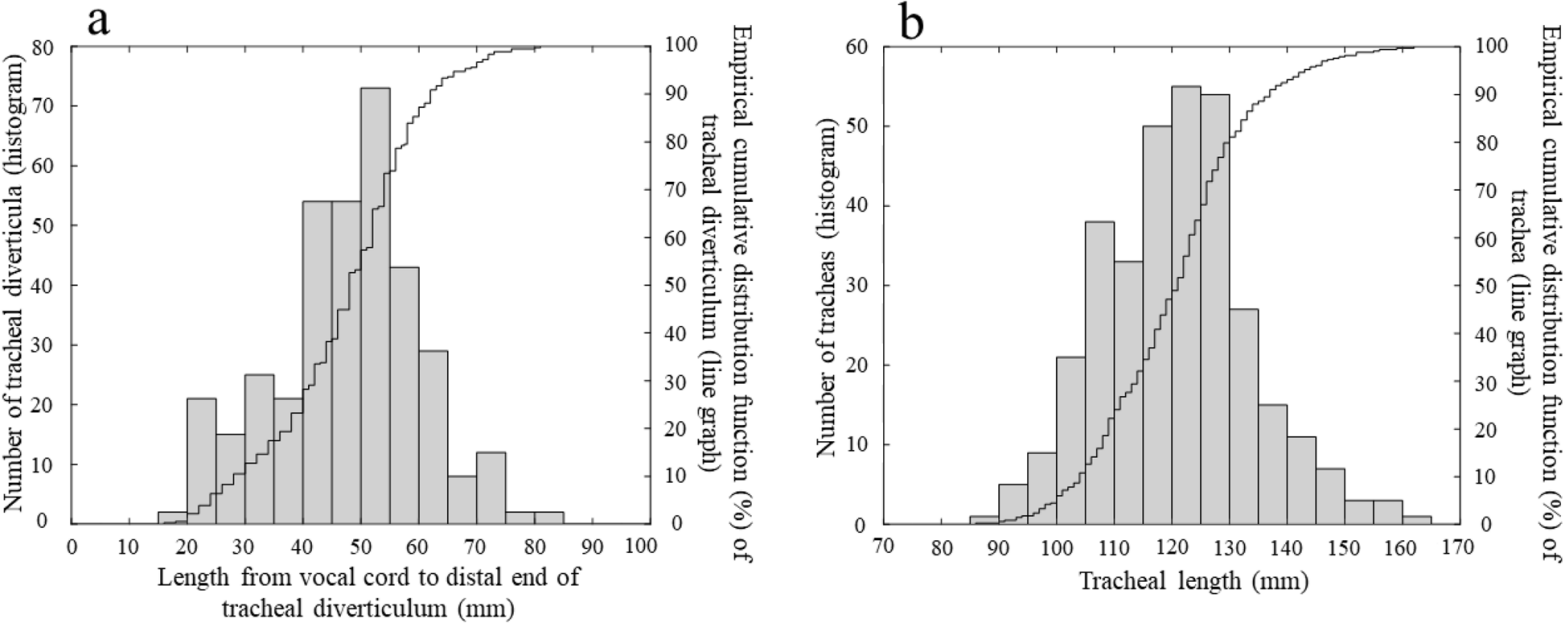
**a** Lengths from the vocal cord to the distal end of tracheal diverticula and its empirical cumulative distribution function. When the tracheal tube was fixed where the line of the vocal cord guide overlapped with the patient’s vocal cord and the tracheal tube’s length from the distal end of the tracheal cuff to the vocal cord guide of was 60 mm (e.g., Parker and Portex products as a 7.0-mm inner diameter), 85.3% of tracheal diverticula were considered to be sealed completely by the tracheal cuff which is located distal to the tracheal diverticula, whereas 14.7% were sealed incompletely and at risk of receiving positive-pressure ventilation. **b** Tracheal length and its empirical cumulative distribution function. When the length from the distal end of the tube to the vocal cord guide was 105 mm (e.g., Shiley products as an inner diameter of 7.0 mm), 12.6% of tracheas were at risk of receiving unintended bronchial intubation

The structural parameters of three single-lumen tracheal tubes with five different inner diameter sizes (6.0–8.0 mm) in Japan are shown in Supplementary Table 2. Each parameter increased in an inner diameter size-dependent manner. Inter-product differences in structural parameters were greater for the length from the distal end of the tracheal cuff to the vocal cord guide than for that from the distal end of the tracheal tube to the distal end of the tracheal cuff (15–20 mm vs 0–5 mm). Figure 4a shows the percentage of tracheal diverticula located distal to the tracheal cuff of the fixed tracheal tube where the line of the vocal cord guide overlapped with the patient’s vocal cord. When the tracheal tube’s length from the distal end of the tracheal cuff to the vocal cord guide was 60 mm (e.g., Parker and Portex products with an inner diameter of 7.0 mm), 14.7% of the tracheal diverticula were thought to be sealed incompletely and were at risk of positive-pressure ventilation during tracheal intubation where the vocal cord guide overlaps with the patient’s vocal cord. When the length from the distal end of the tracheal cuff to the vocal cord guide was 70 mm (e.g., Portex products with an.

inner diameter of 8.0 mm), 4.4% of the tracheal diverticula were thought to be sealed incompletely and were at risk of positive-pressure ventilation. As the length from the distal end of the tracheal cuff to the vocal cord guide increases, the risk of incomplete sealing of tracheal diverticula decreases (Supplementary Table 2).

Figure 4b shows the percentage of tracheas considered to have unintended bronchial intubation. When the length from the distal end of the tube to the vocal cord guide was at least 105 mm (e.g., Shiley products with an inner diameter of 7.0 mm), 12.6% of the tracheas were at risk of receiving unintended bronchial intubation during tracheal intubation where the vocal cord guide overlaps with the patient’s vocal cord. Although tracheal diverticula were less likely to be incompletely sealed with the Shiley tube than with the Parker and Portex tubes because of the Shiley tube’s longer length from the distal end of the tracheal cuff to the vocal cord guide, the Shiley tube also had a longer length from the distal end of the tube to the vocal cord guide than Parker and Portex tubes and was considered to be more likely to cause unintended bronchial intubation.

In summary, tracheal tubes whose length from the distal end of the tracheal cuff to the vocal cord guide was ≥ 70 mm had a low risk of incomplete sealing of tracheal diverticula (< 5%), and tracheal tubes whose length from the distal end of the tube to the vocal cord guide was ≤ 95 mm had a low risk of unintended bronchial intubation (< 5%). No products in this study had a low risk of both outcomes at the same time (Supplementary Table 2). In general, tracheal tubes with a smaller inner diameter had a higher possibility of incomplete sealing of tracheal diverticula and those with a larger inner diameter had a high possibility of unintended bronchial intubation. The Parker tube with an inner diameter of 8.0 mm, Portex tubes with inner diameters of 7.5 and 8.0 mm, and the Shiley tube with an inner diameter of 6.0 mm had relatively lower compound risks of incomplete sealing of tracheal diverticula and unintended bronchial intubation.

## Discussion

The main findings of this study were that (1) single-lumen tracheal tube fixation, where the vocal cord guide overlaps with the patient’s vocal cord, has a risk of incomplete sealing of tracheal diverticula depending on the tracheal tube products and (2) no products in this study satisfied both outcomes of a low risk of incomplete sealing of tracheal diverticula (< 5%) and a low risk of unintended bronchial intubation (< 5%).

Tracheal diverticula were previously considered a rare congenital or acquired condition^1^; however, but because of advances in diagnostic imaging, such as thin-slice CT, recent studies have reported the prevalence of tracheal diverticula to be 0.3%–8.1% and that it is not rare [1, 3, 17–21]. In our study, the prevalence of tracheal diverticula was 5.7%, which is similar to that reported previously [1, 3, 17–21]. According to these reports, it is thought that many tracheal intubation attempts were carried out in patients with tracheal diverticula without the practitioner noticing the existence of tracheal diverticula. In this study, tracheal diverticula were commonly located on the right dorsal side of the trachea at the sternoclavicular joint level, which almost coincided with the level of the second thoracic vertebral body; this finding is consistent with previous findings [1, 2]. The trachea at that level is the transitional point between the extrathoracic and intrathoracic trachea. At this level, the right dorsal wall of the trachea lacks the support of the esophagus and is considered one of the most vulnerable regions in the trachea against positive airway pressure anatomically [2].

In general anesthesia, positive-pressure ventilation for tracheal diverticula could causes tracheal diverticula rupture, subcutaneous emphysema, and pneumomediastinum [7–14]. Thus, tracheal diverticulum is considered as a comorbidity that requires attention in airway management with positive pressure ventilation and preventing positive-pressure ventilation to tracheal diverticula is important to avoid these adverse events. Many of the patients with tracheal diverticula in this study did not have an evident orifice, and the possibility that some patients did not have a tracheal diverticulum but had a paratracheal air cyst could not be ruled out. However, a dynamic CT report has shown that even in paratracheal air cysts without an evident orifice, the cyst contracts during inspiration and expands during expiration, indicating that the many paratracheal air cysts without an evident orifice might be tracheal diverticula and are actually in communication with the trachea [2]. Therefore, the absence of an evident orifice on CT does not mean that there is no diverticulum-tracheal communication. The intra-diverticular pressure during ventilation depends on the diameter of orifice and the existence of a diverticulum-tracheal communication. However, many of the patients in this study did not have large orifice diameters, which may be related to relatively low incidence of injuries related to tracheal intubation and positive pressure ventilation compared to the high prevalence of tracheal diverticulum.

Most tracheal tubes assessed in this study had a risk of incomplete sealing of tracheal diverticula during tracheal intubation with fixation of the tracheal tube where the vocal cord guide overlaps with the patient’s vocal cord. High positive pressure ventilation could widen the diverticulum-tracheal communication that is closed or very small during spontaneous ventilation and could dilate tracheal diverticula, and we therefore consider that all orifices should be sealed or bypassed by the tracheal tube during positive pressure ventilation to avoid positive pressure ventilation into the tracheal diverticula. However, the possibility that paratracheal air cyst without evident orifice has hidden orifices and the possibility that tracheal diverticulum with evident orifice has other hidden orifices cannot be ruled out [2]. Therefore, to prevent positive pressure ventilation to the tracheal diverticulum during positive pressure ventilation in a clinical setting, it is important to seal and bypass not only the obvious orifice but also the entire tracheal diverticulum by the tracheal tube because the positional relationships between all of the orifices, including hidden orifices, and the trachea are not known.

Preventing the incomplete sealing of tracheal diverticula involves complete sealing by the tracheal cuff by fixing the tube deeply where the tracheal cuff is located completely distal to the tracheal diverticula [15]. This method needs deeper tube fixation than it would be in accordance with the vocal cord guide and has a risk of unintended bronchial intubation [22–24]. Although the best way to avoid these risks is to measure the patient’s tracheal length and the characteristics of the tracheal diverticula, to select the most appropriate tracheal tube and inner diameter size, and to use a bronchoscope for confirming no bronchial intubation during general anesthesia in all cases, this requires a significant effort and is impractical. We presented the indications for low-risk tracheal tubes and tube fixation in reference to incomplete sealing of tracheal diverticula and unintended bronchial intubation. When the tube was fixed deeper than in accordance with the vocal cord guide, a length from the distal end of the tracheal cuff to the patient’s vocal cord of ≥ 70 mm had a low risk of incomplete sealing of tracheal diverticula. When the tube was fixed shallower than in accordance with the vocal cord guide, a length from the distal end of the tube to the patient’s vocal cord of ≤ 95 mm had a low risk of unintended bronchial intubation. These indications are beneficial for anesthesiologists when deciding the type and fixation depth of tracheal tubes. Although this was a single-center study performed in Japan and all patients were Asian, the mean tracheal length was similar to that in other reports from Japan and those from other countries, such as England, New Zealand, and East Asian countries [25–30], and the mean tracheal length might be similar across populations. Thus, the characteristics and positional relationships between a tracheal diverticulum and the tracheal tube in this study could be extrapolated to other populations.

Taken together, anesthesiologists might have to consider individual structural parameters of the trachea and tracheal diverticula and choose appropriately sized tracheal tubes to avoid the compound risk of incomplete sealing of tracheal diverticula and unintended bronchial intubation in patients with tracheal diverticula. The results and indications in our study may help anesthesiologists make these decisions.

This study has several limitations. First, this study did not assess other tracheal lesions. Bronchial diverticula are some of the most identified tracheal outpouching lesions whose prevalence were reported to be 21.6%–45.5%; most bronchial diverticula are located at the carina and main bronchi [31–33]. Although almost all bronchial diverticula are located at the distal end of the tracheal tube in the tracheal intubation and numerous bronchial diverticula have received positive-pressure ventilation to date, no study has reported the rupture or other issues of bronchial diverticula related to tracheal intubation. Thus, the impact of positive-pressure ventilation on bronchial diverticula might be lower than that on tracheal diverticula. Second, this study did not assess the classification of tracheal diverticulum as congenital or acquired and the changes in tracheal diverticula over time or the direct impact of positive-pressure ventilation to tracheal diverticula that were sealed incompletely. Further research is needed to investigate the natural course of tracheal diverticula and their changes after general anesthesia. Third, other types of tracheal tubes, which were not assessed in this study, may have different structural parameters and positional relationships between tracheal diverticula and the tracheal tube. Forth, this study is an observational and simulation study using thoracic CT. In the general anesthesia, tracheal length may change according to the patient’s position (e.g., lateral position or head-down tilt) and patient’s neck position (anteflexion or retroflexion). Despite these limitations, to the best of our knowledge, this study is the first to investigate the positional relationships between tracheal diverticula and the tracheal tube with tracheal intubation during general anesthesia. Anesthesiologists perform tracheal intubation routinely. Thus, the present findings raise the importance of caution regarding incomplete sealing of tracheal diverticula and the risk of positive-pressure ventilation to tracheal diverticula.

## Conclusion

In conclusion, single-lumen tracheal tube fixation, where the vocal cord guide overlaps with the patient’s vocal cord, is associated with risk of incompletely sealing of tracheal diverticula depending on the tube’s manufacturer and tube’s inner diameter size, although it was not a high risk. The use of small inner diameter sized tube relative to patient’s body size is high risk of incomplete sealing of tracheal diverticula.

### Supplementary Information

Additional file 1: **Supplementary Table 1.** Age-stratified prevalence of tracheal diverticula. **Supplementary Table 2.** Structural parameters of tracheal tubes of each inner diameter size. **Supplementary Figure 1.** Tracheal tubes compared in this study. (a) Parker Flex-Tip (Parker Medical, Bridgewater, CT, USA). (b) Portex Soft Seal (ICU Medical, San Clemente, CA, USA). (c) Shiley TaperGuard (Medtronic, Dublin, Ireland).

## Data Availability

The datasets used and analyzed during the current study are available from the corresponding author on reasonable request.

## Abbreviations

CI: Confidence interval
CT: Computed tomography
IRB: Institutional Review Board
SD: Standard deviation
UMIN: University hospital Medical Information Network
