# Supplementary material for: Positional relationships between a tracheal diverticulum and the tracheal tube under general anesthesia: a single-center observational and simulation study
Source: BMC Anesthesiol. 2023 Nov 25;23:386. doi: 10.1186/s12871-023-02347-y (PMC10675875; doi:10.1186/s12871-023-02347-y)
Supplement: Supplementary file 1 — Additional file 1: Supplementary Table 1. Age-stratified prevalence of tracheal diverticula. Supplementary Table 2. Structural parameters of tracheal tubes of each inner diameter size. Supplementary Figure 1. Tracheal tubes compared in this study. (a) Parker Flex-Tip (Parker Medical, Bridgewater, CT, USA). (b) Portex Soft Seal (ICU Medical, San Clemente, CA, USA). (c) Shiley TaperGuard (Medtronic, Dublin, Ireland). [file 12871_2023_2347_MOESM1_ESM.docx]

**Positional relationships between a tracheal diverticulum and the tracheal tube under general anesthesia: a single-center observational and simulation study**

Kunihiro Mitsuzawa. M.D. ^1, 2*^, Tsukasa Kumagai. M.D. ^2^, Haruo Uchida. M.D. ^2^, Toshiyuki Shimizu. M.D. ^2^

^1^ Department of Anesthesiology and Resuscitology, Shinshu University School of Medicine, 3-1-1, Asahi, Matsumoto City, Nagano 390-8621, Japan

^2^ Department of Anesthesiology, Nagano Prefectural Shinshu Medical Center, 1332, Suzaka, Suzaka City, Nagano, 382-8577, Japan

***Corresponding author:** Kunihiro Mitsuzawa

Department of Anesthesiology and Resuscitology, Shinshu University School of Medicine

3-1-1, Asahi, Matsumoto City, Nagano 390-8621, Japan

Tel.: +81 263 37 2670; Fax: +81 263 35 2734

Email: [mitsukuni@shinshu-u.ac.jp](mailto:mitsukuni@shinshu-u.ac.jp)

| **Supplementary Table 1.** Age-stratified prevalence of tracheal diverticula | | | | |
| --- | --- | --- | --- | --- |
| Age group (years) | Patients with tracheal diverticula (male/female) | Patients without tracheal diverticula (male/female) | Total number of patients (male/female) | Age-stratified prevalence (%) |
| 0–10 | 0 (0/0) | 5 (2/3) | 5 (2/3) | 0.0 |
| 11–20 | 1 (0/1) | 52 (38/14) | 53 (38/15) | 1.9 |
| 21–30 | 7 (4/3) | 127 (73/54) | 134 (77/57) | 5.2 |
| 31–40 | 11 (8/3) | 213 (130/83) | 224 (138/86) | 4.9 |
| 41–50 | 21 (7/14) | 386 (212/174) | 407 (219/188) | 5.2 |
| 51–60 | 38 (17/21) | 500 (270/230) | 538 (287/251) | 7.1 |
| 61–70 | 50 (22/28) | 930 (520/410) | 980 (542/438) | 5.1 |
| 71–80 | 99 (46/53) | 1388 (807/581) | 1487 (853/634) | 6.7 |
| 81–90 | 80 (36/44) | 1392 (669/723) | 1472 (705/767) | 5.4 |
| Over 91 | 26 (12/14) | 528 (163/365) | 554 (175/379) | 4.7 |
| All ages | 333 (152/181) | 5521 (2,884/2,637) | 5854 (3,036/2,818) | 5.7 |
| Values are presented as number or proportion (%). | | | | |

| **Supplementary Table 2.** Structural parameters of tracheal tubes of each inner diameter size. | | | | | | |
| --- | --- | --- | --- | --- | --- | --- |
|  |  | Inner diameter size (mm) | | | | |
|  | Products | 6.0 | 6.5 | 7.0 | 7.5 | 8.0 |
| Distal end of tube-to-distal end of cuff (mm) | Parker | 25 | 25 | 30 | 30 | 35 |
|  | Portex | 25 | 25 | 30 | 30 | 30 |
|  | Shiley | 25 | 30 | 30 | 30 | 30 |
| Distal end of tube-to-proximal end of cuff (mm) | Parker | 55 | 60 | 65 | 65 | 70 |
|  | Portex | 55 | 55 | 60 | 60 | 60 |
|  | Shiley | 60 | 65 | 65 | 70 | 75 |
| Distal end of cuff-to-vocal cord guide (mm) | Parker | 60 | 60 | 60 | 60 | 65 |
|  | Portex | 55 | 60 | 60 | 65 | 70 |
|  | Shiley | 75 | 75 | 75 | 80 | 85 |
| Distal end of tube-to-vocal cord guide (mm) | Parker | 85 | 85 | 90 | 90 | 100 |
|  | Portex | 80 | 85 | 90 | 95 | 100 |
|  | Shiley | 100 | 105 | 105 | 110 | 115 |
| Possibility of incomplete sealing of tracheal diverticula (%) | Parker | 14.7 | 14.7 | 14.7 | 14.7 | 6.6 |
|  | Portex | 26.6 | 14.7 | 14.7 | 6.0 | 4.4 |
|  | Shiley | 1.1 | 1.1 | 1.1 | 0.0 | 0.0 |
| Possibility of unintended bronchial intubation (%) | Parker | 0.0 | 0.0 | 0.6 | 0.6 | 6.0 |
|  | Portex | 0.0 | 0.0 | 0.6 | 1.8 | 6.0 |
|  | Shiley | 6.0 | 12.6 | 12.6 | 24.0 | 34.5 |
| Parker: Parker Flex-Tip, Portex: Portex Soft Seal, Shiley: Shiley Taperguard. Values are number (mm) or proportion (%). | | | | | | |


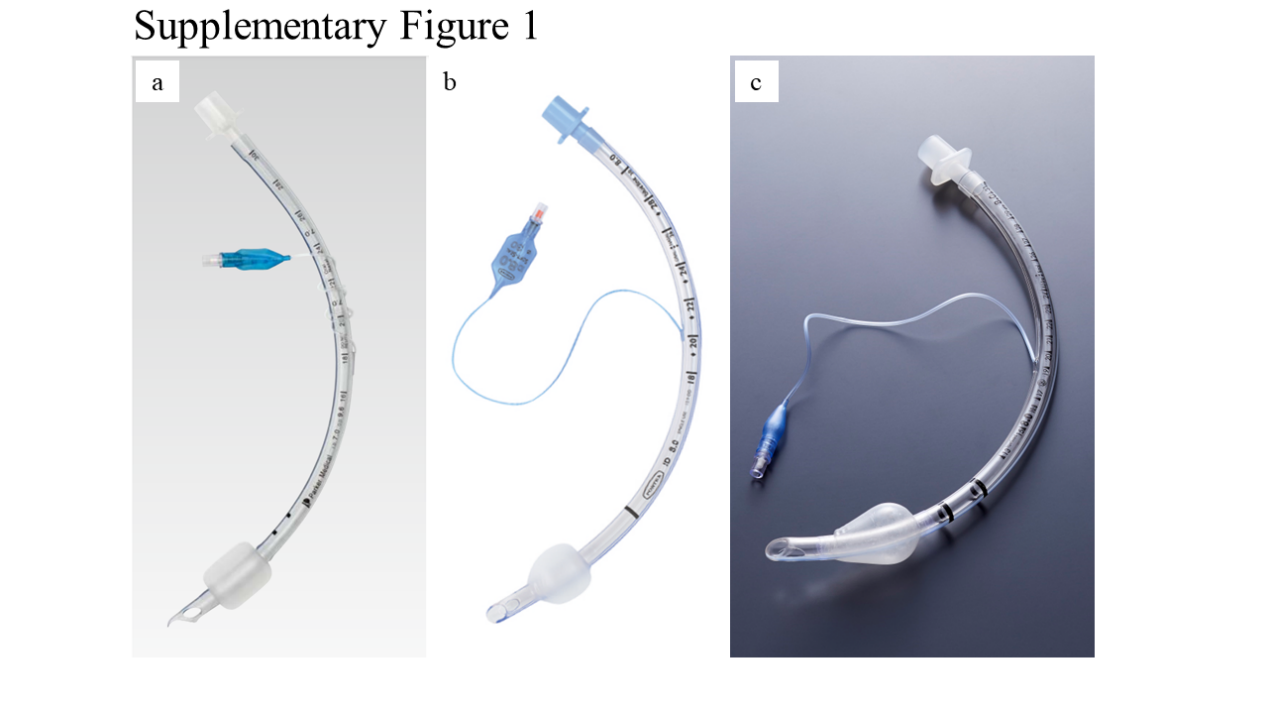


**Supplementary Figure 1.** Tracheal tubes compared in this study. (a) Parker Flex-Tip (Parker Medical, Bridgewater, CT, USA). (b) Portex Soft Seal (ICU Medical, San Clemente, CA, USA). (c) Shiley TaperGuard (Medtronic, Dublin, Ireland).
